# The BCL-2 selective inhibitor ABT-199 sensitizes soft tissue sarcomas to proteasome inhibition by a concerted mechanism requiring BAX and NOXA

**DOI:** 10.1038/s41419-020-02910-2

**Published:** 2020-08-24

**Authors:** Alina Muenchow, Sandra Weller, Clemens Hinterleitner, Elke Malenke, Stefanie Bugl, Stefan Wirths, Martin R. Müller, Klaus Schulze-Osthoff, Walter E. Aulitzky, Hans-Georg Kopp, Frank Essmann

**Affiliations:** 1 Department of Molecular Oncology, Robert Bosch Centre for Tumor Diseases and Robert-Bosch-Hospital, Auerbachstr. 110, 70376 Stuttgart, Germany; 2 Dr. Margarete-Fischer-Bosch Institute of Clinical Pharmacology and University of Tübingen, Auerbachstr. 112, 70376 Stuttgart, Germany; 3 Department of Internal Medicine II, University of Tübingen, Otfried-Müller-Str. 10, 72076 Tübingen, Germany; 4 University Children’s Hospital Tübingen, Hoppe-Seyler-Str. 1, 72076 Tübingen, Germany; 5 Department of Oncology, Hematology and Immunology, Klinikum Siloah, 30449 Hannover, Germany; 6 Department of Molecular Medicine, Interfaculty Institute for Biochemistry, University of Tübingen, Hoppe-Seyler-Str. 4, 72076 Tübingen, Germany; 7 German Cancer Consortium (DKTK) and German Cancer Research Center (DKFZ), 69120 Heidelberg, Germany; 8 Department of Hematology, Oncology and Palliative Medicine, Robert-Bosch-Hospital, Auberbachstr. 110, 70376 Stuttgart, Germany

**Keywords:** Cancer therapeutic resistance, Sarcoma, Apoptosis, Stress signalling, Targeted therapies

## Abstract

Soft tissue sarcomas (STS) are a heterogeneous group of malignancies predominantly affecting children and young adults. Despite improvements in multimodal therapies, 5-year survival rates are only 50% and new treatment options in STS are urgently needed. To develop a rational combination therapy for the treatment of STS we focused on ABT-199 (Venetoclax), a BCL-2 specific BH3-mimetic, in combination with the proteasome inhibitor bortezomib (BZB). Simultaneous inhibition of BCL-2 and the proteasome resulted in strongly synergistic apoptosis induction. Mechanistically, ABT-199 mainly affected the multidomain effector BAX by liberating it from BCL-2 inhibition. The combination with BZB additionally resulted in the accumulation of BOK, a BAX/BAK homologue, and of the BH3-only protein NOXA, which inhibits the anti-apoptotic protein MCL-1. Thus, the combination of ABT-199 and BZB sensitizes STS cells to apoptosis by simultaneously releasing several defined apoptotic restraints. This synergistic mechanism of action was verified by CRISPR/Cas9 knock-out, showing that both BAX and NOXA are crucial for ABT-199/BZB-induced apoptosis. Noteworthy, efficient induction of apoptosis by ABT-199/BZB was not affected by the p53 status and invariably detected in cell lines and patient-derived tumor cells of several sarcoma types, including rhabdomyo-, leiomyo-, lipo-, chondro-, osteo-, or synovial sarcomas. Hence, we propose the combination of ABT-199 and BZB as a promising strategy for the treatment of STS, which should warrant further clinical investigation.

## Introduction

Soft tissue sarcomas (STS) comprise a heterogeneous group of malignancies of mesenchymal origin with more than 100 different histologic subtypes and entities^1^. Sarcomagenesis arises from various alterations, such as fusion proteins resulting from genomic rearrangements, mutations in tumor-suppressor and cell cycle regulatory genes, and DNA copy-number variations. Sarcomas are traditionally classified in two major genetic groups: the first group includes sarcomas with simple karyotypes and specific genetic alterations like *EWS-ATF1* fusions in Ewing sarcomas, *KIT* mutations in GISTs, or *PAX3-FKHR* fusions in alveolar rhabdomyosarcomas. The second group is represented by complex genetic aberrations associated with impaired p53 checkpoint function, dysfunctional DNA repair by homologous or nonhomologous end joining, and an increase of unrepaired double-strand breaks. Complex karyotypes in STS are frequently related to *BRAF, RAS* (H-, K-, and N-), *PTEN*, *RB1*, *BRCA2*, *APC*, and *PIK3CA* mutations^2^.

Due to the genetic heterogeneity of STS, especially in a metastatic disease, therapeutic options are limited and the average 5-year survival rate, regardless of the type of sarcoma, is only 50%^3^. A commonly used chemotherapeutic treatment option especially for young patients is doxorubicin that shows a rather steep dose-response curve^4^. Because resistance to monotherapies frequently occurs in sarcomas, the combined treatment with doxorubicin and ifosfamide or additional anti-cancer drugs is thought to overcome drug resistance. In several clinical trials such combination therapies showed promising improvements in progression-free survival (PFS). However, the 5-year survival rate was only marginally affected^5^ and, due to its high toxicity, doxorubicin in combination with ifosfamide is a rational treatment option only in young and fit patients. Other combinations of doxorubicin, e.g., with the HSP-90 inhibitor 17-DMAG and the histone deacetylase inhibitor vorinostat, have been shown to synergistically increase cell death in cultured small cell sarcomas^6^. Efficacy of vorinostat in the treatment of sarcoma has also been tested together with the proteasome inhibitor bortezomib (BZB), but despite good tolerability patients did not respond in a clinical phase II trial^7^. In line, although proteasome inhibition was recently shown to be effective in the treatment of Ewing sarcoma^8^, BZB was ineffective in other STS^9^. Thus, efficient treatment of sarcomas still remains an unmet clinical need^10^.

A relatively new class of targeted anti-cancer drugs that are efficient especially in the treatment of hematopoietic malignancies are BH3-mimetics/BCL-2 inhibitors^11^. BH3-mimetics are small molecule drugs that - like BH3-only proteins (e.g. BAD, BID, BIM, NOXA, and PUMA) – specifically bind to anti-apoptotic proteins of the BCL-2 family (BCL-2, BCL-xL, BCL-w, MCL-1, and A1). The BCL-2 inhibitors occupy the hydrophobic cleft in anti-apoptotic proteins that physiologically accommodates the BH3-domain of pro-apoptotic proteins. Thereby, BH3-mimetics prevent binding of pro-apoptotic BH3-only or multidomain effector proteins (BAX, BAK, and BOK). Ultimately, the apoptosis effectors are no longer counteracted by anti-apoptotic proteins, and active conformers of BAX, BAK, and BOK are free to oligomerize and permeabilize the mitochondrial outer membrane. Permeabilization releases cytochrome c into the cytosol and initiates activation of cell-death-specific proteases, the caspases, that establish apoptotic demise of the cell^12^.

Like BH3-only proteins, the pro-apoptotic multidomain effector proteins specifically interact with anti-apoptotic BCL-2 proteins. Although interaction of BOK with anti-apoptotic proteins is debated^13,14^, the binding preferences of BAK and BAX are largely established. BAK preferentially binds to MCL-1 and BCL-xL^15^, while BAX binds to BCL-2, BCL-xL, and BCL-w^16^. Thus, the prototypic and broad BH3-mimetic ABT-737 can induce cell death in various tumor cell types, but has dose-limiting side effects such as thrombocytopenia^17^. A successor of ABT-737 is the orally available BH3-mimetic ABT-199 (Venetoclax) that shows enhanced specificity for BCL-2. ABT-199 induces cell death in several hematopoietic malignancies and is also tested in other cancer entities. In line with its specific binding to BCL-2, ABT-199 tends to be more efficient in multiple myeloma (MM) with high expression of BCL-2 and less effective in MCL-1 overexpressing MM^18^. This limitation might be overcome by combination of ABT-199 with the newly developed MCL-1 specific inhibitors, such as A-1210477 and others^19,20^.

Despite sequence homology to the pore-forming effector proteins BAX and BAK, the protein BOK has only recently been verified as a bona fide pro-apoptotic multidomain protein of the BCL-2 family^21^. BOK, similar to BAX and BAK, is an apoptosis effector that mediates mitochondrial outer membrane permeabilization (MOMP)^21,22^. The regulation of BOK-induced apoptosis is rather unconventional, because BOK shows “intrinsic instability” or “metastability”, meaning that BOK spontaneously changes its conformation from an inactive to an active state^23^. How active BOK is prevented from apoptosis induction is rather unclear. Although inhibition of BOK by anti-apoptotic BCL-2 proteins, such as MCL-1, has been repeatedly shown^13,14,21^, a recent publication proposed that BOK does neither interact with anti-apoptotic BCL-2 proteins nor requires activation by BH3-only proteins^22^. Rather, BOK activity is controlled at the level of protein stability and is prevented from constitutive apoptosis induction by the AMFR/gp78 E3 ubiquitin ligase complex that targets BOK to proteasomal degradation. Consequently, inhibition of BOK degradation by the proteasome inhibitor MG132 was shown to stabilize BOK and induce apoptosis, while knock-down of BOK attenuates MG132-induced apoptosis^22^. Interestingly, proteasome inhibitors have also been reported to induce expression of the pro-apoptotic BH3-only protein NOXA^24^. NOXA specifically binds and inactivates the anti-apoptotic proteins MCL-1 and A1^25^ that in turn predominantly counteract the effectors BAK^26–28^ and allegedly BOK^13,14^.

Intrigued by proteasome inhibitor mediated stabilization of BOK^22^ and the reported induction of NOXA expression^29^, we hypothesized that the combined inhibition of the proteasome and anti-apoptotic BCL-2 proteins by BH3-mimetics should synergize in apoptosis induction. Indeed, we show here that the combined application of ABT-199 and the proteasome inhibitor BZB synergistically induces apoptosis in STS cell lines and primary tumor-derived cells irrespective of their tissue origin. As the underlying mechanism we identified that the drug combination not only inhibited BCL-2 but also enhanced expression of BOK and the MCL-1 antagonist NOXA. As a net result, BCL-2 and MCL-1 are simultaneously liberated from their prebound pro-apoptotic BCL-2 proteins. In line with the proposed mechanism, CRISPR/Cas9-mediated knock-out of *BAX*, but not *BAK* or *BOK*, significantly reduced apoptosis induction by ABT-199/BZB. Moreover, the additional knock-down of *NOXA* in *BAX*-deficient cells most efficiently blocked apoptosis induction by ABT-199/BZB. We propose that the combined treatment with ABT-199/BZB is a promising strategy to improve the therapy of various STS types independent of their tissue origin.

## Results

Tumor cells frequently overexpress anti-apoptotic BCL-2 proteins and have therefore become “BCL-2 addicted”^30^. BCL-2 addicted cells are prone to apoptosis induction by small-molecule inhibitors of anti-apoptotic BCL-2 proteins, such as ABT-199^31^. In addition to directly blocking anti-apoptotic proteins, tumor cells can also be sensitized toward apoptosis by shifting the equilibrium of BCL-2 proteins^32^ towards pro-apoptotic members, such as BH3-only or multidomain effector proteins. Because expression of the BH3-only protein NOXA as well as the pro-apoptotic effector BOK^22^ is enhanced by proteasome inhibition, we hypothesized that reducing the anti-apoptotic capacity by ABT-199 and simultaneously enhancing the pro-apoptotic activity of NOXA and BOK by the proteasome inhibitor BZB might be a rational strategy in STS.

### ABT-199/BZB synergistically reduce viability

**Fig. 1:**
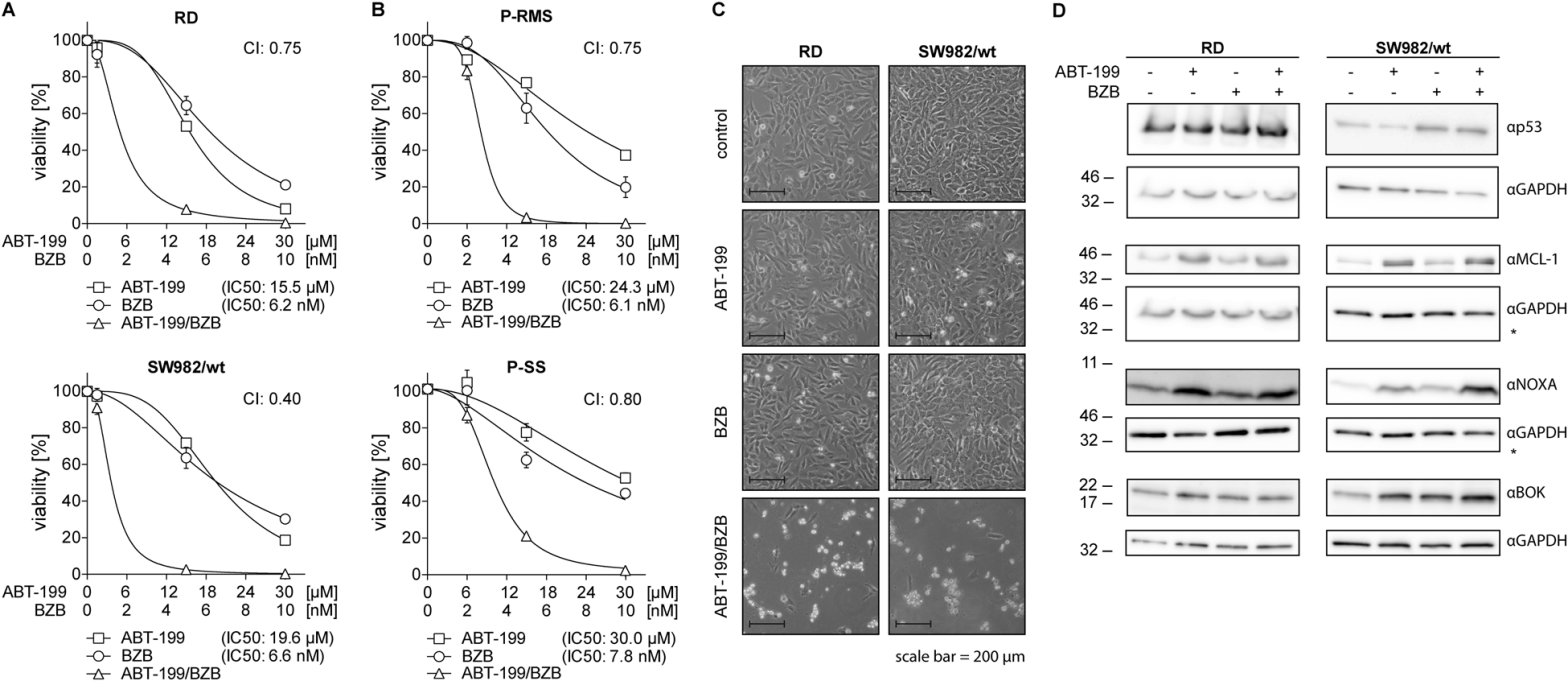
Synergistic reduction of cell viability and enhanced BOK and NOXA expression by combined treatment with ABT-199 and BZB. **a**, **b** RD, SW982, and corresponding patient-derived tumor cells were incubated with the indicated concentrations of ABT-199 and/or BZB for 48 h. Cell viability was assessed by CellTiter-Glo Luminescent assay and normalized to the DMSO vehicle control. Drug interaction and synergisms were analyzed by calculation of the combination index (CI) using CompuSyn software. **c** Microscopic assessment of RD and SW982 shows only marginal numbers of floating (dead) cells 24 h after incubation with 5 nM BZB or 15 µM ABT-199 alone. In contrast, ABT-199/BZB massively induces cell death. **d** RD and SW982 cells were incubated in the absence (ctrl) or presence of 5 nM BZB, 15 µM ABT-199, or both for 8 h in the presence of Q-VD-OPh, before expression of the indicated proteins was analyzed by western blotting. Asterisk represents identical GAPDH blot because identical membrane was probed.

Initially, we performed dose-response viability assays in RD (rhabdomyosarcoma) and SW982 (synovial sarcoma) cells with mutant and wildtype p53, respectively. Cell viability of RD and SW982 cells was assayed after 48 h of incubation with various concentrations of ABT-199 and/or BZB. In both cell lines single drug treatments led to a moderate decrease in cell viability (Fig. 1a). The combination of BZB and ABT-199 was however strongly synergistic, as also confirmed by calculation of the combination index. Importantly, similar synergistic activity was seen in primary tumor cells derived from patients with rhabdomyosarcoma (P-RMS) and synovial sarcoma (P-SS), respectively (Fig. 1b). Also, microscopic evaluation confirmed that neither ABT-199 nor BZB alone elicited significant toxicity, while the combination of both drugs acted in concert to decrease cell viability (Fig. 1c).

To investigate the mechanism of the combined BCL-2 protein and proteasome inhibition in more detail, we analyzed expression of BOK, NOXA, and p53 by western blotting after incubation of cells with ABT-199 and/or BZB (Fig. 1d). p53 mutant RD cells revealed a high constitutive expression of p53, while p53 was upregulated by the proteasome inhibitor in p53wt SW982 cells. BZB augmented expression of BOK and NOXA as well as of the NOXA-inhibitory protein MCL-1 that is acceptedly degraded by the proteasome^33,34^ (Fig. 1d). Intriguingly, also ABT-199 treatment alone led to the accumulation of BOK, NOXA, and MCL-1. Moreover, although more pronounced in SW982 and to a lesser extent also in RD cells, ABT-199/BZB strongly augmented the expression of BOK, NOXA, and MCL-1 (Fig. 1d).

### ABT-199/BZB induce apoptotic cell death

**Fig. 2:**
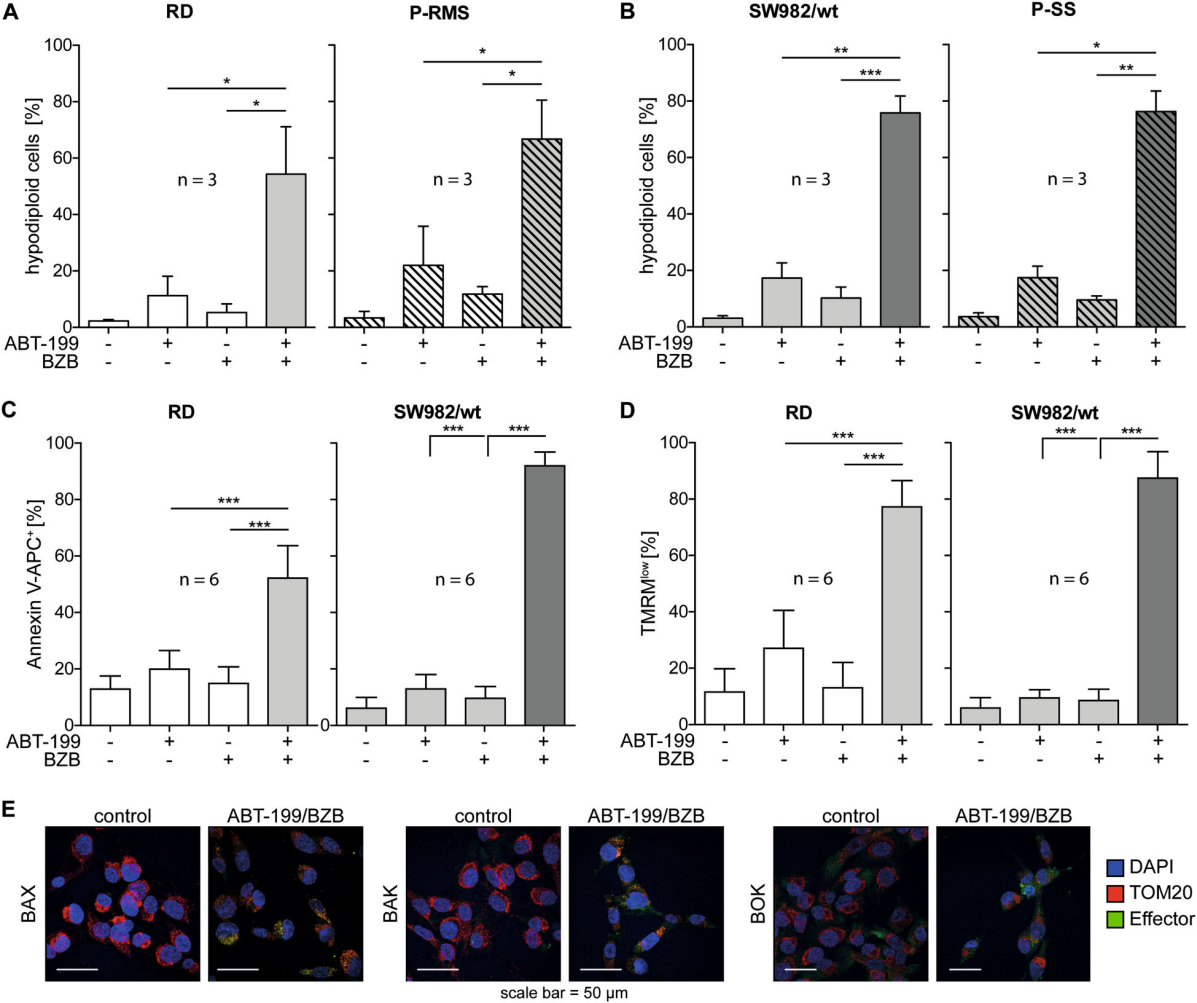
ABT-199/BZB-induced cell death is apoptotic. **a**, **b** RD and SW982 cells were incubated in the presence of 5 nM BZB and 15 µM ABT-199 alone or in combination. After 48 h the percentage of cells with hypodiploid DNA (subG1) was assessed by flow cytometry. **c**, **d** RD and SW982 cells were incubated with 5 nM BZB and/or 15 µM ABT-199 for 24 h. Subsequently, exposure of phosphatidylserine (Annexin V) and loss of mitochondrial membrane potential (TMRM) were analyzed by flow cytometry. **e** SW982 cells were incubated without or with ABT-199/BZB for 24 h. Immunofluorescence microscopy shows activation/clustering of the effectors BAX, BAK, and BOK (green) in response to ABT-199/BZB.

Intrigued by the efficient cell death induction by ABT-199/BZB, we thought to analyze the mode of cell death in more detail. RD and SW982 cells and corresponding primary tumor cells, i.e. P-RMS and P-SS, were treated with ABT-199 and BZB alone or in combination for 24 h. Subsequently, cell nuclei were stained with propidium iodide and relative DNA content was analyzed by flow cytometry; cells with hypodiploid DNA (subG1) were assumed apoptotic^35^. In these analyses <22% of hypodiploid cells were detected after incubation with BZB or ABT-199 alone in each sarcoma cell line and tumor-derived cells. In contrast, simultaneous incubation with ABT-199/BZB-induced apoptosis in 54–76% of the cells (Fig. 2a, b).

These results are in line with the proposed hypothesis that inhibition of BCL-2 and the proteasome synergistically induce cell death. To corroborate that cell death is apoptotic we analyzed exposure of phosphatidylserine (PS) and loss of ΔΨ_m_, two established hallmarks of apoptosis, by staining cells with fluorescence-labeled Annexin V or TMRM. In line with the previous results, <20% of RD and SW982 cells exposed PS after incubation with ABT-199 or BZB alone, while >50% (RD) and >90% (SW982) Annexin V^+^ cells were detected after incubation with the drug combination (Fig. 2c and Supplemental Fig. 2A). Compared to the single drug treatments, the proportion of cells exhibiting reduced mitochondrial membrane potential was also significantly higher after simultaneous incubation with ABT-199 and BZB, i.e. 77% in RD and 87% in SW982 cells (Fig. 2d, Supplemental Fig. 2B). Thus, the combination of ABT-199 and BZB induces cell death by apoptosis, as evidenced by DNA fragmentation, exposure of PS and loss of mitochondrial membrane potential. Finally, fluorescence microscopy revealed clustering and, hence, activation of BAX, BAK, and BOK after incubation with ABT-199/BZB (Fig. 2e) that was not detected in cells incubated with BZB or ABT-199 alone (Supplemental Fig. 1).

### Specific synergism of BZB with BCL-2 inhibition

**Fig. 3:**
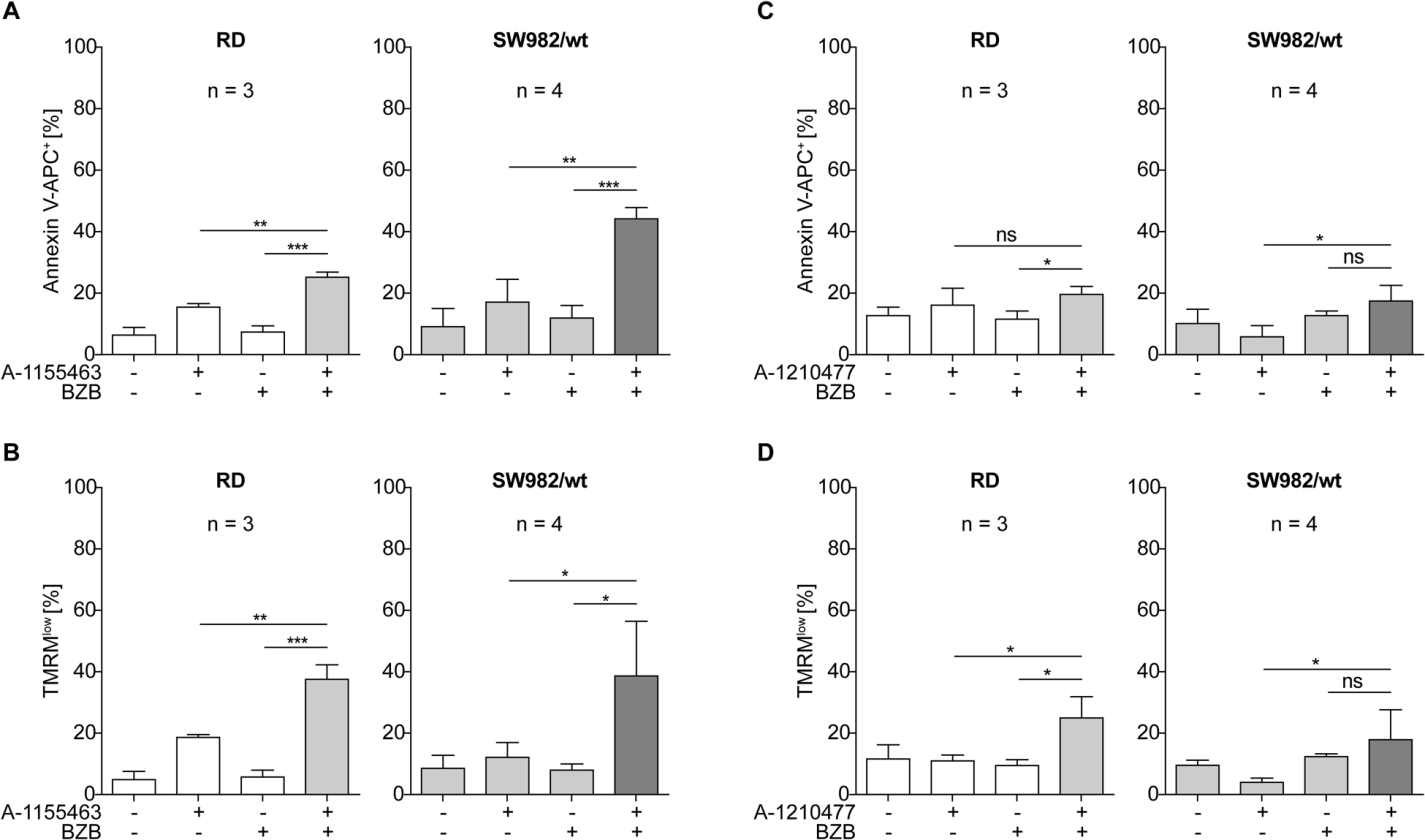
Specific synergism of BZB with BCL-2 inhibition. RD and SW982 cells were incubated with 5 nM BZB and/or 15 µM of the BCL-xL inhibitor A-1155463 (**a**, **b**) or 15 µM of the MCL-1 inhibitor A-1210477 (**c**, **d**). After 24 h, exposure of phosphatidylserine (**a**, **c**) and loss of mitochondrial membrane potential (**b**, **d**) were analyzed by flow cytometric staining with Annexin V and TMRM, respectively.

To delineate the role of anti-apoptotic BCL-2 proteins in ABT-199/BZB-induced apoptosis, we further utilized recently developed BCL-xL and MCL-1 specific inhibitors. RD and SW982 cells were incubated with the BCL-xL inhibitor A-1155463^36^ or the MCL-1 specific inhibitor A-1210477^20^ alone or in combination with BZB. After 24 h cells were flow cytometrically analyzed for apoptosis induction. Similar to ABT-199, A-1155463, and A-1210477, when applied alone, elicited only marginal exposure of PS or loss of mitochondrial membrane potential in ~20% of RD or SW982 cells (Fig. 3a–d). However, compared to ABT-199, the combination of the BCL-xL inhibitor A-1155463 and BZB resulted in reduced Annexin V^+^ and TMRM^low^ cells, with <45% of RD and SW982 being apoptotic (Fig. 3a, b). The MCL-1 inhibitor A-1210477 and BZB even less efficiently induced apoptosis with <20% of cells positive for PS exposure (Fig. 3c) and <25% of cells showing low ΔΨ_m_ (Fig. 3d). Hence, the synergism of BZB and ABT-199 is clearly based on specific inhibition of BCL-2 rather than of BCL-xL or MCL-1.

### BAX is a key effector of ABT-199/BZB-induced apoptosis

**Fig. 4:**
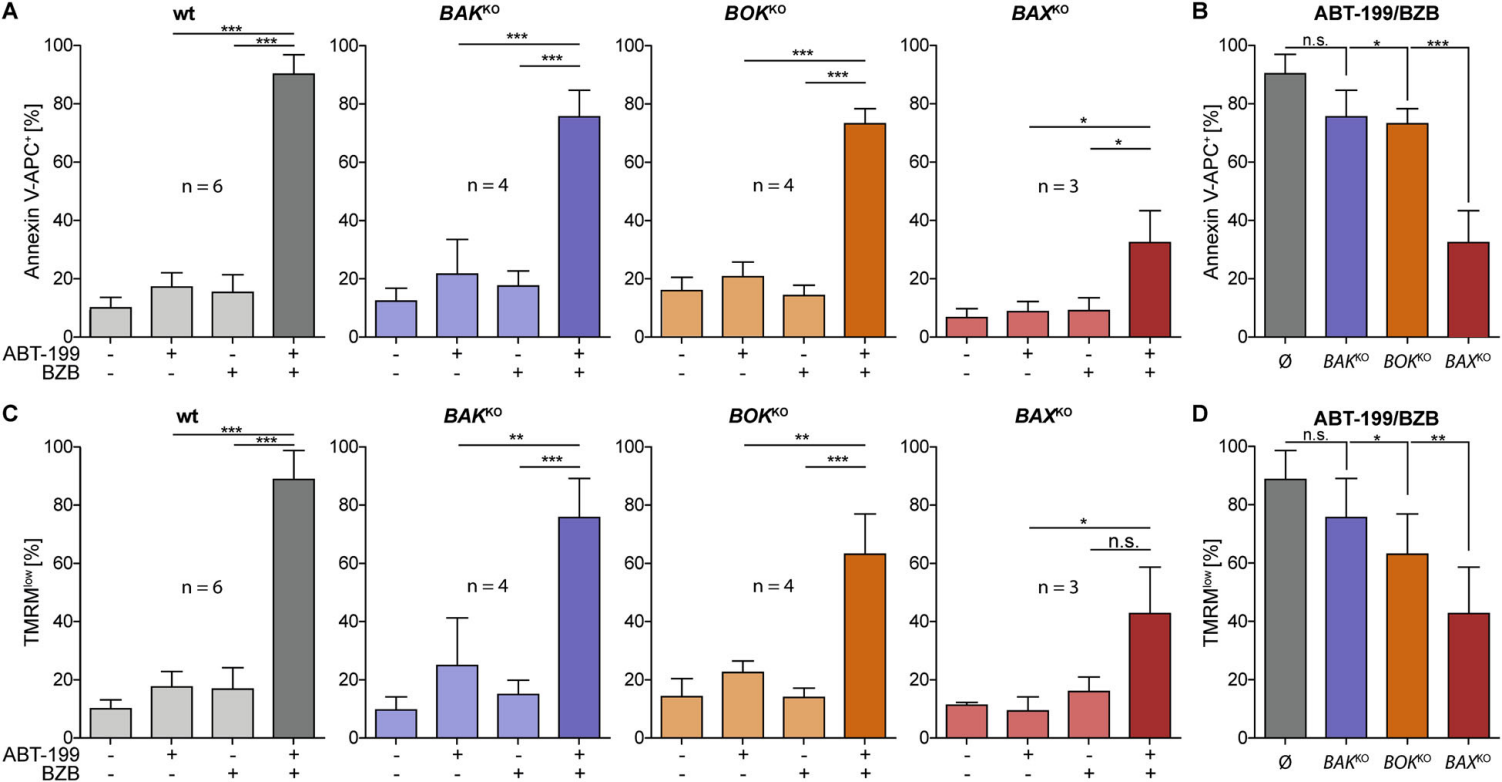
BAX is a key effector of ABT-199/BZB-induced apoptosis. SW982 knock-out cells deficient in the effector proteins BAX, BAK, or BOK were generated by CRISPR/Cas9 technology and incubated with 5 nM BZB and/or 15 µM ABT-199 for 24 h. Apoptosis induction was evaluated by flow cytometric measurements of phosphatidylserine exposure using Annexin V (**a**, **b**) or of the mitochondrial membrane potential using TMRM (**c**, **d**).

Though these data identify BCL-2 rather than BCL-xL or MCL-1 as the anti-apoptotic BCL-2 protein that is most relevant for the synergism with proteasome inhibition, we next explored the identity of the pro-apoptotic effector BCL-2 protein responsible for the release of cytochrome c from mitochondria. In view of the complex interplay of BCL-2 family proteins and the redundant function of effector proteins in MOMP, it is likely that the synergism is based on a set rather than one single effector protein. To elucidate the relevance of the known effectors BAX, BAK, and BOK, we generated SW982 cell lines devoid of each of these proteins by CRISPR/Cas9-mediated KO technology. As verified by western blotting (Supplemental Fig. 3), we selected clonal cell lines with individual knock-outs of *BAX*, *BAK*, or *BOK*, which retained similar expression of other BCL-2 family proteins. These cell lines were incubated with ABT-199/BZB and analyzed for apoptosis by flow cytometry. ABT-199/BZB-induced Annexin V^+^ (Fig. 4a, b) and TMRM^low^ (Fig. 4c, d) cells, which was similar in the parental wt, *BAK* or *BOK* knock-out SW982 cells. Interestingly, however, in SW982/*BAX*^KO^ cells apoptosis induction and loss of mitochondrial membrane potential by the drug combination were most strongly reduced (Fig. 4a, c). Thus, the synergistic apoptosis induction by inhibition of BCL-2 largely depends on BAX rather than on BAK or BOK. This is in line with the weak sensitization of BZB-challenged cells by inhibitors of BCL-xL or MCL-1 (Fig. 3) as well as the fact of BCL-2 being the main BAX antagonist^28^.

### NOXA contributes to synergistic cell death induction by ABT-199/BZB

**Fig. 5:**
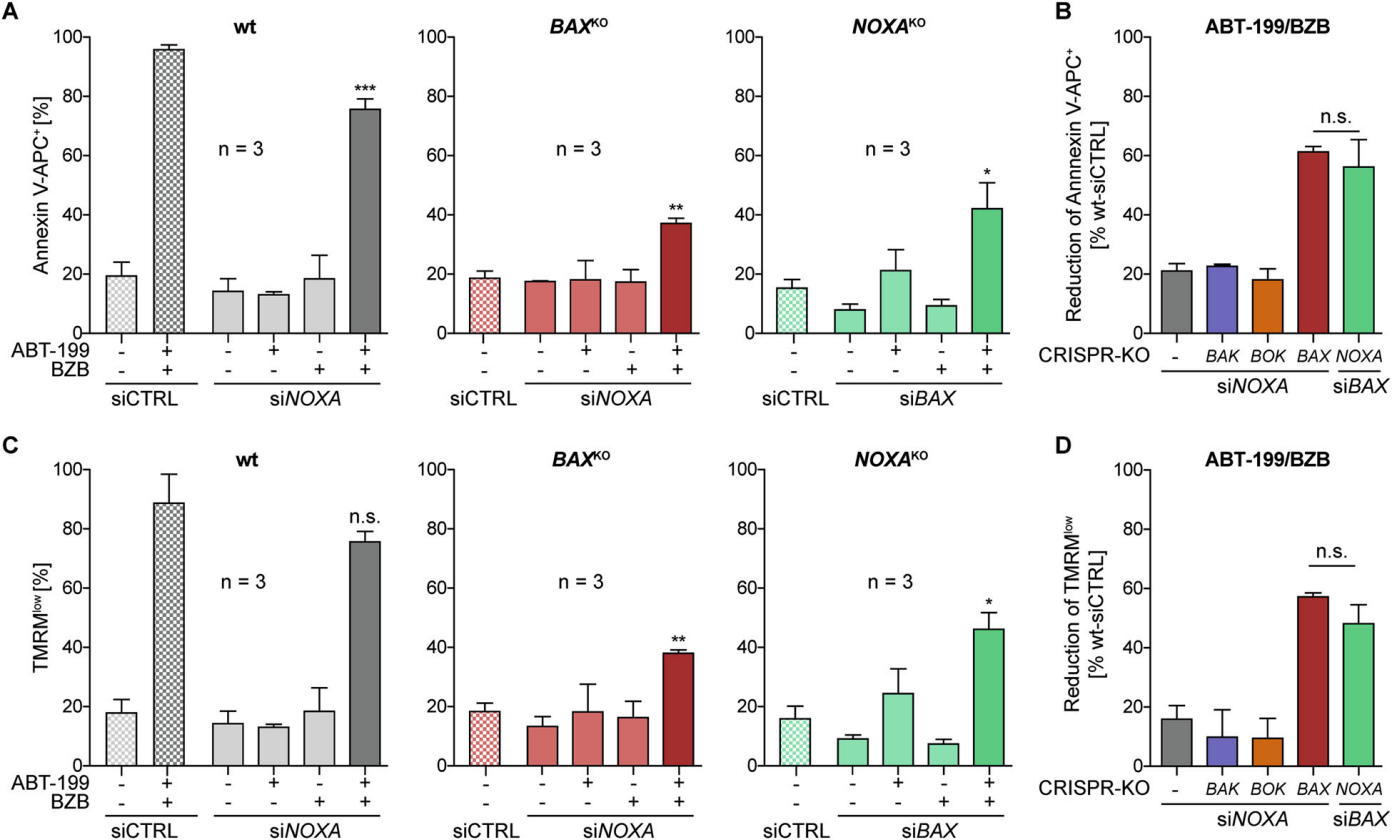
Both NOXA and BAX mediate synergistic apoptosis induction by ABT-199/BZB. SW982/wt and SW982/BAX^KO^ or SW982/NOXA^KO^ were transfected with siRNA-*NOXA* or siRNA-*BAX* and incubated with 5 nM BZB and/or 15 µM ABT-199. Apoptosis induction was evaluated after 24 h by measuring **a** phosphatidylserine exposure (Annexin V-APC^+^) and **c** loss of mitochondrial membrane potential (TMRM^low^). **b**, **d** Relative reduction of phosphatidylserine exposure (**b**) and loss of mitochondrial membrane potential (**d**) by siRNA-mediated knock-down of *NOXA* and *BAX* in wt and *BAK*-, *BOK*-, or *BAX*-deficient SW982 cells in response to treatment with ABT-199/BZB.

Data so far shows that the synergistic activity of ABT-199/BZB largely relies on the BCL-2/BAX axis. Because BZB enhances expression of NOXA that inhibits the BAK/BOK antagonist MCL-1, we further aimed to pinpoint the role of NOXA in the synergistic apoptosis induction. Hence, we transfected SW982/wt and different KO cell lines with siRNA to knock-down expression of *NOXA* and subsequently analyzed apoptosis induction by ABT-199/BZB. In wt, *BAK*^KO^ and *BOK*^KO^ SW982 cells knock-down of *NOXA* had little impact on apoptosis induction, and ~80% of the cells were apoptotic in each cell line after incubation with ABT-199/BZB (Supplemental Fig. 4A, B). In contrast, in SW982/*BAX*^KO^ cells knock-down of *NOXA* diminished cell death induction to 40% as compared to 80% in SW982/wt cells (Fig. 5a, c). Similar results were obtained in an inverse approach by knocking down *BAX* in SW982/*NOXA*^KO^ cells (Fig. 5a, c and Supplemental Fig. 4C). Thus, the data corroborates the hypothesis that NOXA, by blocking MCL-1, targets a BCL-2/BAX-independent axis of apoptosis regulation. In combination, inhibition of BCL-2 and NOXA together are instrumental for synergistic cell death induction by ABT-199/BZB.

### ABT-199/BZB is effective in various sarcoma cell lines and tumor-derived cells

**Fig. 6:**
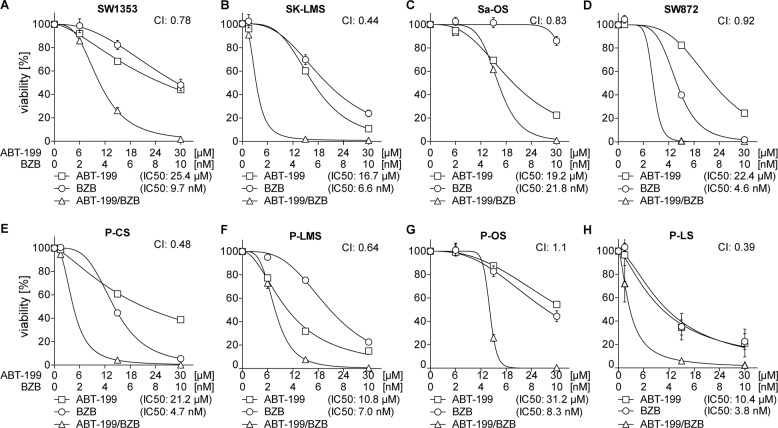
ABT-199/BZB synergistically abolish cell viability in tumor cells of different STS types. **a**–**d** Sarcoma cell lines and **e**–**h** primary tumor-derived cells of different STS types were incubated in the presence of the indicated concentrations of BZB, ABT-199, or both. After 48 h cell viability was analyzed by CellTiter-Glo Luminescent cell viability assay and normalized to the DMSO control. Viability curves, IC50, and CI values were calculated using GraphPad Prism and CompuSyn. BZB and ABT-199 synergism is indicated by CI values <1. P-CS primary chondrosarcoma, P-LMS primary leiomyosarcoma, P-OS primary osteosarcoma, P-LS primary liposarcoma.

**Fig. 7:**
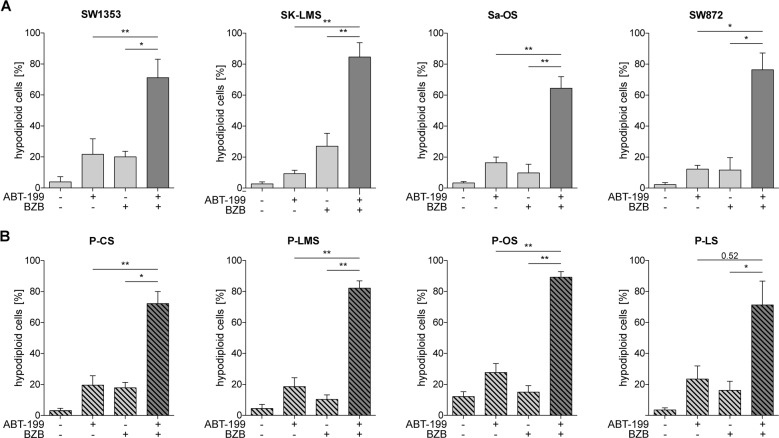
Enhanced cell death induction by ABT-199/BZB in established and tumor-derived sarcoma cell lines. Cell lines and primary tumor-derived cells of different STS types (Fig. 6) were additionally treated with 5 nM BZB in the presence or absence of 15 µM ABT-199 for 48 h. After 48 h flow cytometric staining with propidium iodide was used to determine the percentage of cells with hypodiploid DNA (subG1).

Although data in RD and SW982 cells show that ABT-199 and BZB synergistically induce cell death regardless of the status of p53, there are typically varying genetic aberrations in soft tissue sarcomas. To account for the genetic heterogeneity, we analyzed additional STS cell lines for their response to ABT-199/BZB. We found that combined application of ABT-199/BZB effectively and in most cases synergistically reduced viability of STS cell lines, i.e., SW1353 (chondrosarcoma; CI: 0.78), SK-LMS (leiomyosarcoma; CI: 0.44), Sa-OS (osteosarcoma; CI: 0.83), and SW872 (liposarcoma; CI: 0.92; Fig. 6a–d). These data indicate that the synergistic action of ABT-199/BZB is largely independent of the genetic background of sarcoma cell lines (Supplemental Table 1). Moreover, synergistic cell death induction was found in patient-derived primary cells of different STS types (Fig. 6e–h). In line with the reduced viability, we detected significantly more hypodiploid (subG1) cells in each of the cell lines (Fig. 7a) and primary tumor cells (Fig. 7b) in response to incubation with ABT-199/BZB. Thus, these findings suggest that the combined use of ABT-199 and BZB is a promising strategy for the treatment of soft tissue sarcomas, irrespective of their tissue origin and p53 status.

## Discussion

The mitochondrial apoptosis signaling pathway is regulated by BCL-2 family proteins. The BCL-2 multidomain effector proteins BAX, BAK, and BOK mediate permeabilization of the mitochondrial outer membrane by pore formation. The pore formation by BAX and BAK relies on an activating conformational change that might occur spontaneously^15,37,38^ or after transient interaction with BH3-only proteins^39,40^. The active conformers are then either inhibited by anti-apoptotic BCL-2 proteins or accumulate in pore-forming multimers. In contrast to BAX and BAK, BOK shows intrinsic instability^41^ and a significant proportion of BOK proposedly is in its active conformation. Thus, to prevent constitutive apoptosis induction, BOK is broadly controlled by proteasomal degradation^22^. Also, expression of the MCL-1 inhibiting pro-apoptotic BH3-only protein NOXA is enhanced by inhibition of the proteasome^42,43^.

Given this information we hypothesized that the combined action of ABT-199 and BZB, i.e., enhanced expression of the MCL-1 antagonist NOXA and stabilization of the effector BOK together with simultaneous inhibition of anti-apoptotic BCL-2, exacerbates apoptosis by releasing restraints on BAX, BAK, and BOK. In line with this hypothesis and the proteasomal degradation of BOK^22^, we show enhanced expression of NOXA and BOK in RD and SW982 cells incubated with ABT-199/BZB. The expression of NOXA and BOK (Fig. 1d) and efficient apoptosis induction by the combined presence of ABT-199/BZB is therefore well compatible with the above-mentioned hypothesis. Furthermore, we provide evidence that this drug combination efficiently induces cell death in several STS cell lines (RD, SW982, SW1353, SK-LMS, Sa-OS, and SW872). Because these STS cell lines differ in their genetic alterations and tissue origin, we propose that the therapeutic strategy is more widely applicable in the treatment of advanced STS. This proposition is corroborated by effective apoptosis induction in cells derived from STS tumor samples: irrespective of their genetic alterations also cells derived from patients effectively undergo apoptosis in response to ABT-199/BZB.

BZB had already been tested earlier in the treatment of STS in a dose escalation study but was found to be largely ineffective^9^. Similarly, the combined treatment of BZB with histone deacetylase inhibitor vorinostat, while being well tolerated, mostly failed to show responses in a phase I study in advanced malignancies. In contrast, we here combined BZB with the BCL-2 inhibitor ABT-199. ABT-199 is effective in the treatment of hematopoietic malignancies^11^. Both drugs are in clinical use and well tolerated, suggesting that the combination of both drugs is a suitable therapeutic option for the treatment of STS. Especially in view of the limited treatment options for STS our findings warrant further clinical investigation. Therefore, it is conceivable that the combination of ABT-199/BZB is also applicable in patients that cannot receive high-dose doxorubicin-based therapy.

CRISPR/Cas9-mediated knock-out of *BAX* reveals that BAX is most relevant for synergistic action of ABT-199/BZB. Also, as compared to ABT-199, the specific inhibition of BCL-xL or MCL-1 does not synergize with BZB. This is in line with ABT-199 targeting the BCL-2/BAX-axis while BZB independently targets the MCL-1/BAK&BOK-axis by enhancing expression of NOXA and BOK (Supplemental Fig. 5).

ABT-199 is effective in CLL, AML, non-Hodgkin lymphoma^11,44–46^, and likely in multiple myeloma with t11;14 translocation^18^. Interestingly, acquired resistance to ABT-199 in AML cell lines is overcome by the MCL-1 specific inhibitor VU661013, which synergizes with ABT-199 in apoptosis induction^47^. The synergistic activity of ABT-199 and VU661013 is well compatible with the here proposed mechanism for the synergism of ABT-199 and BZB. Proteasome inhibition acceptedly enhances expression of the MCL-1 inhibiting BH3-only protein NOXA^42,48^. Originally, NOXA (*PMAIP1*) has been described as a p53-induced BH3-only protein^49^, which is in line with stronger induction of NOXA expression in p53-proficient SW982 compared to p53-defective RD cells. Regardless of the specific mechanism of enhanced NOXA expression, e.g. enhanced p53-dependent or independent^50^ transcription or reduced proteasomal degradation^42,43^, the net result is inhibition of anti-apoptotic MCL-1. In summary, the proposed strategy inactivates the BAX antagonist BCL-2 and the BAK antagonist MCL-1. Thus, the anti-apoptotic capacity of cells to restrain BAX and BAK is largely reduced.

Additionally, BZB-mediated block of BOK degradation shifts the BCL-2 rheostat even further towards cell death. The shift towards apoptosis still prevails even when considering the BZB-induced increase of MCL-1 expression, because also expression of NOXA is enhanced. In this scenario apoptosis is augmented irrespective of whether BOK interacts with anti-apoptotic BCL-2 proteins^13,14^ or not^22,51^. Although interaction of BOK with anti-apoptotic BCL-2 proteins is debated, stabilization of BOK and MCL-1 might even be caused upstream of the proteasome by interaction of BOK with MCL-1. Indeed, BOK and MCL-1 have been proposed to interact at least functionally and constitute a regulatory feedback^52^. Non-proteasomal regulation of BOK expression by mRNA stability, as proposed by Oyneagucha et al.^52^, was also published by Fernandez-Marrero et al.^53^. Thus, there is increasing evidence that there are levels of regulation for the expression of BOK other than proteasomal degradation.

In this study we propose the combination of BZB and ABT-199 for the treatment of patients with soft tissue sarcoma. The data in the present study is well in line with analog approaches to treat patients with relapsed/refractory multiple myeloma (MM). A phase Ib trial (NCT1794507) and an ongoing phase III trial (NCT02755597) indicate promising results for the combined treatment of MM patients with ABT-199 and BZB^54,55^. In another trial (NCT01794520), the benefit from ABT-199/BZB for relapsed/refractory MM patients was associated with high BCL-2 expression (i.e. BCL-2/BCL-xL or BCL-2/MCL-1 ratio): 12 out of the 14 patients with t(11:14) translocation, which correlates with enhanced BCL-2 expression, showed a positive response^18^. In general, the combination of specific BCL-2 inhibitors with other approved anti-cancer drugs repeatedly proved a successful strategy in various tumor models^56,57^). The data presented here suggests that BCL-2 inhibition by BH3-mimetics is also a valid option for the treatment of solid tumors, especially in combination with established and effective drugs^56,58–62^.

## Materials and methods

### Tumor-derived cells and STS cell lines

Patients from the University Tübingen Center for Soft Tissue Sarcomas and Bone Tumors who underwent surgical resection of primary or metastatic tumors were included in this study (Supplementary Table 2). All patients had given written informed consent to the scientific analysis of tissue samples, the study was approved by the ethics committee of the University of Tübingen. Fresh tumor samples were obtained after surgical resection. Primary sarcoma samples were mechanically dissociated into 1–2 mm^3^ tissue fragments, washed, and cultured in standard media containing 1% Antibiotic-Antimycotic (Gibco, Life Technologies, Darmstadt, Germany). All cells and cell lines were cultured in media (DMEM, DMEM/F12, or MEM; Gibco) supplemented with 10% fetal calf serum (FCS; Biochrom) and 1% Antibiotic-Antimycotic. MEM was additionally supplemented with 1% Na-pyruvate and 1% glutamine. Human STS cell lines including SW982, SK-LMS-1, SW872, RH30/RD, SW1353, and Sa-OS were authenticated by STR-profiling at the DSMZ and ATCC and cultured in medium as stated in Supplemental Table 2. Cells were harvested after incubation in trypsin/EDTA solution, centrifuged at 600 × *g* for 5 min and processed for subsequent analysis as described.

### Viability assay

Cell viability was assessed using CellTiter-Glo Assay (Promega, Mannheim, Germany) according to the manufacturer’s instructions. Briefly, 4000 cells per well were seeded in white 96-well plates with plastic bottom (Invitrogen, Karlsbad, Germany). After 2 h BZB (0.5–10 nM; Selleckchem, Munich, Germany) and ABT-199 (1.5–30 μM; Selleckchem) were added. Cell viability was analyzed in quadruples after 48 h.

### Flow cytometry

#### Annexin V and TMRM staining

Cells were harvested using trypsin/EDTA, resuspended in supernatant and washed in ice-cold PBS. Then, pellets were resuspended in 300 μL Annexin V-binding buffer (PBS, 2.5 mM CaCl_2_) supplemented with Annexin V-APC (1:100) and incubated for 20 min. Subsequently, samples were analyzed using a FACS Lyric flow cytometer. To assess the mitochondrial membrane potential, cell pellets were resuspended in PBS supplemented with 2% FCS and 50 nM of the potentiometric dye TMRM. Cells were incubated at 37 °C for 20 min and fluorescence was analyzed using a FACS Lyric flow cytometer. The proportion of TMRM^low^ and Annexin V^+^ cells was calculated using FACS Suite software.

#### Nicoletti assay

Cells were harvested by trypsination, washed in ice-cold PBS, and resuspended in ice-cold hypotonic buffer N (0.1% sodium citrate, 0.1% Triton X-100, and 50 µg/mL propidium iodide) as described^63^. After 30 min of incubation at 4 °C relative cellular DNA content was detected using a Becton Dickinson FACS Calibur flow cytometer (Becton Dickinson, Heidelberg, Germany) and Cell Quest Software. Cells showing relative DNA content <2N were assumed apoptotic.

### Antibodies

The following antibodies were used: BAX (Cell Signaling, #2772), BAK (Cell Signaling, #3814), BOK (Abcam, #186745), BCL-2 (Cell Signaling, #15071), MCL-1 (Cell Signaling, #5453), NOXA (Merck, #OP180), p53 (Santa Cruz, #sc-126), GAPDH (Cell Signaling, #2118), TOM20 (Becton Dickinson, #612278). Secondary anti-mouse (#7076S), and anti-rabbit (#7074S) horseradish-coupled antibodies were from Cell Signaling. Chicken anti-rabbit Alexafluor488 and goat anti-mouse DyeLight 649 were from Thermo Fisher Scientific (Waltham, MA, USA) and Abcam (Berlin, Germany), respectively.

### Western blot

Cells were harvested by scraping, washed in ice-cold PBS, and lysed in 150 μL/10 cm^2^ lysis buffer (50 mM Tris-HCl pH 7.6, 250 mM NaCl, 0.1% Triton X-100, and 5 mM EDTA) supplemented with protease and phosphatase inhibitors (complete and PhosphoSTOP, Roche, Basel, Switzerland). Samples were sonified (Diagenode, Liège, Belgium) and cleared by centrifugation (15 min, 4 °C, 14000 × *g*). Relative protein content of supernatants was assessed using Pierce BCA Protein Assay Kit (Thermo Fisher Scientific). Equal amounts of protein (typically 30 μg) were separated by SDS-PAGE and blotted (Biometra FastblotTM, Analytic Jena, Jena, Germany) onto nitrocellulose membrane (0.1 μm; GE Healthcare, Munich, Germany) by semi-dry blotting (1 mA/cm^2^, 1 h). Primary antibody was applied in PBST (0.1% Tween-20) overnight at 4 °C. Membranes were washed thrice 10 min incubated with horseradish-coupled secondary antibody (1:2000) for 2 h at room temperature. After washing, ECL solution was applied (SuperSignal™ West Dura, Thermo Fisher Scientific) and specific bands were detected using a Stella gel documentation system (Raytest Isotopenmessgeräte GmbH, Straubenhardt, Germany).

### Fluorescence microscopy

SW982 cells were seeded on coverslips (#1) in 12-well plates at a density of 5 × 10^4^/well. The next day, medium was removed and cells were incubated in medium supplemented with 10 µM Q-VD-OPh and the indicated drugs. After 24 h, cells were fixed in PBS/4% formaldehyde for 30 min on ice, washed in PBS, and incubated overnight at 4 °C with the following primary antibodies in PBS/4% BSA/0.05% Saponin: mouse anti-TOM20 (clone 29, BD Biosciences), anti-BAK-NT (06–536, Merck Millipore), anti-BAX-NT (06–499, Merck Millipore), or anti-BOK (EPR15331, Abcam). Samples were washed in PBS and incubated with fluorescence-labeled secondary antibodies (chicken anti-rabbit Alexafluor488, Life Technologies and goat anti-mouse DyeLight649, Abcam) for 4 h. Then, cells were washed in PBS supplemented with 100 ng/mL DAPI for 5 min, washed twice in PBS and mounted using DAKO fluorescent mounting medium (Life Technologies). Fluorescence microscopy images were taken using a Leica TCS SP8 confocal laser scanning microscope. Images (512 × 512 pixel) were taken using excitation at 405 nm, 488 nm, and 647 nm at 600 Hz scan speed with line average and identical laser intensity/amplification of detectors for each series.

### CRISPR/Cas9 Knock-out

For lentivirus production 1 × 10^7^ HEK2983FT cells/flask were transfected with 10 μg pMD2.G, 15 μg psPAX2, and 20 μg plentiCRISPRv2 containing the desired guideRNA^64^ using PEI (polyethylenimine 40,000; Warrington, PA, USA) reagent (1 mg/mL PEI in 25 mM HEPES pH 7.5 and 150 mM NaCl). Virus containing supernatant was collected 48 h and 72 h after transfection and concentrated by ultracentrifugation. SW982 cells were transduced with 10 μL concentrated virus overnight and then cultured with 1 μg/mL puromycin (Thermo Fisher Scientific) for 48 h. For the generation of knock-out cell lines deficient in *BAK*, *BOK*, *BAX*, or *NOXA*, clones were isolated and protein expression was analyzed by western blotting.

### RNA interference

0.6 × 10^5^ cells/well were seeded in 12-well plates 24 h prior to transfection. Then, cells were transfected with ON-TARGET Plus Smartpool siRNAs targeting *BAX* or *NOXA* or non-targeted (NT) Smartpool ON-TARGET plus control siRNA (Horizon Discovery, Waterbeach, UK) using Dharmafect Ι reagent (Thermo Fisher Scientific) according to the manufacturer’s protocol. After 24 h cells were incubated with ABT-199 and/or BZB for additional 24 h. Then, cells were harvested and analyzed by flow cytometry. For verification of knock-down efficacy, cells were lysed and assessed through western blot analysis as mentioned previously.

### Statistical analysis

Continuous variables are presented as mean ± SD and categorical variables are given by number and percentages. The statistical significance of differences was analyzed using Student’s *t* test. All statistical tests were considered significant when *p* < 0.05. Statistical analyses were computed using GraphPad Prism (v5.04) or Microsoft Excel (Professional Plus 2019).

## Supplementary information

Supplemental Figure Legends

Supplemental Figure 1

Supplemental Figure 2

Supplemental Figure 3

Supplemental Figure 4

Supplemental Figure 5

Supplemental Tables 1&2
