# Supplementary material for: The BCL-2 selective inhibitor ABT-199 sensitizes soft tissue sarcomas to proteasome inhibition by a concerted mechanism requiring BAX and NOXA
Source: Cell Death Dis. 2020 Aug 24;11(8):701. doi: 10.1038/s41419-020-02910-2 (PMC7445285; doi:10.1038/s41419-020-02910-2)
Supplement: Supplementary file 1 — Supplemental Figure Legends [file 41419_2020_2910_MOESM1_ESM.docx]

Supplemental Figure Legends

Supplemental Figure 1: ABT-199/BZB induces clustering of BAX, BAK, and BOK. SW982/wt cells were incubated with 15 µM ABT-199 and/or 5 nM BZB for 24 h. Fluorescence microscopy of fixed cells shows clustered appearance of BAX, BAK, and BOK at the mitochondria (TOM20) typical for apoptotic cell death exclusively after incubation with ABT-199/BZB. Either drug alone does not elicit cluster formation.

Supplemental Figure 2: ABT-199/BZB synergize to induce exposure of phosphatidyl serine and loss of mitochondrial membrane potential. SW982/wt and RD cells were incubated with or without ABT-199, BZB or both for 24 h. Exposure of phosphatidyl serine and mitochondrial membrane potential were assessed by staining cells with Annexin V-APC and TMRM, respectively. Data was acquired by flow cytometric analysis.

Supplemental Figure 3: Specific knock-out of *BAX*, *BAK* or *BOK* and similar expression of BCL-2 and MCL-1 in CRISPR/Cas9 knock-out cell lines. SW982/wt cells were transduced with lentiviruses carrying Cas9 and sgRNA for *BAX*, *BAK* or *BOK*. Puromycin-resistant clonal cell lines were incubated with or without ABT-199, BZB or both for 8 h in the presence of Q-VD-OPh. Cell lysates were analyzed for the expression of the indicated proteins. Identical blots for GAPDH are shown when the same membranes were re-probed with the different antibodies.

Supplemental Figure 4: Neglectable impact of NOXA knock-down on ABT‑199/BZB-mediated apoptosis in SW982/*BAK*^KO^ and SW982/*BOK*^KO^. A, B) SW982 sublines were transfected with si*NOXA* and apoptosis induction, indicated by A) PS exposure and B) loss of mitochondrial membrane potential, by ABT-199 and/or BZB was analyzed by flow cytometry. C) SW982/wt cells were transduced with CRISPR/Cas9-sg*NOXA* and puromycin-resistant clonal cell line was incubated with or without ABT-199 and BZB alone or in combination for 8 h. Western Blot indicates comparable regulation of BOK expression and knock-out of *NOXA*.

Supplemental Figure 5: Mechanism of synergistic apoptosis induction by ABT-199 and BZB. A) In vital cells BAX oligomerization is prevented by BCL-2, whereas Mcl-1 inhibits BAK and BOK. Proteasomal degradation of BOK is a further mechanism preventing BOK oligomerization. B) BZB blocks proteasomal degradation of BOK and NOXA, allowing oligomerization of BOK and inhibition of MCL-1, respectively. Inhibition of MCL-1 allows oligomerization of BAK, while ABT-199-mediated inhibition of BCL-2 allows oligomerization of BAX. Thus, the break on all three effectors, BAX, BAK and BOK, is released and apoptosis proceeds unconstrained.
