# Supplementary figures and images for: The BCL-2 selective inhibitor ABT-199 sensitizes soft tissue sarcomas to proteasome inhibition by a concerted mechanism requiring BAX and NOXA
Source: Cell Death Dis. 2020 Aug 24;11(8):701. doi: 10.1038/s41419-020-02910-2 (PMC7445285; doi:10.1038/s41419-020-02910-2)

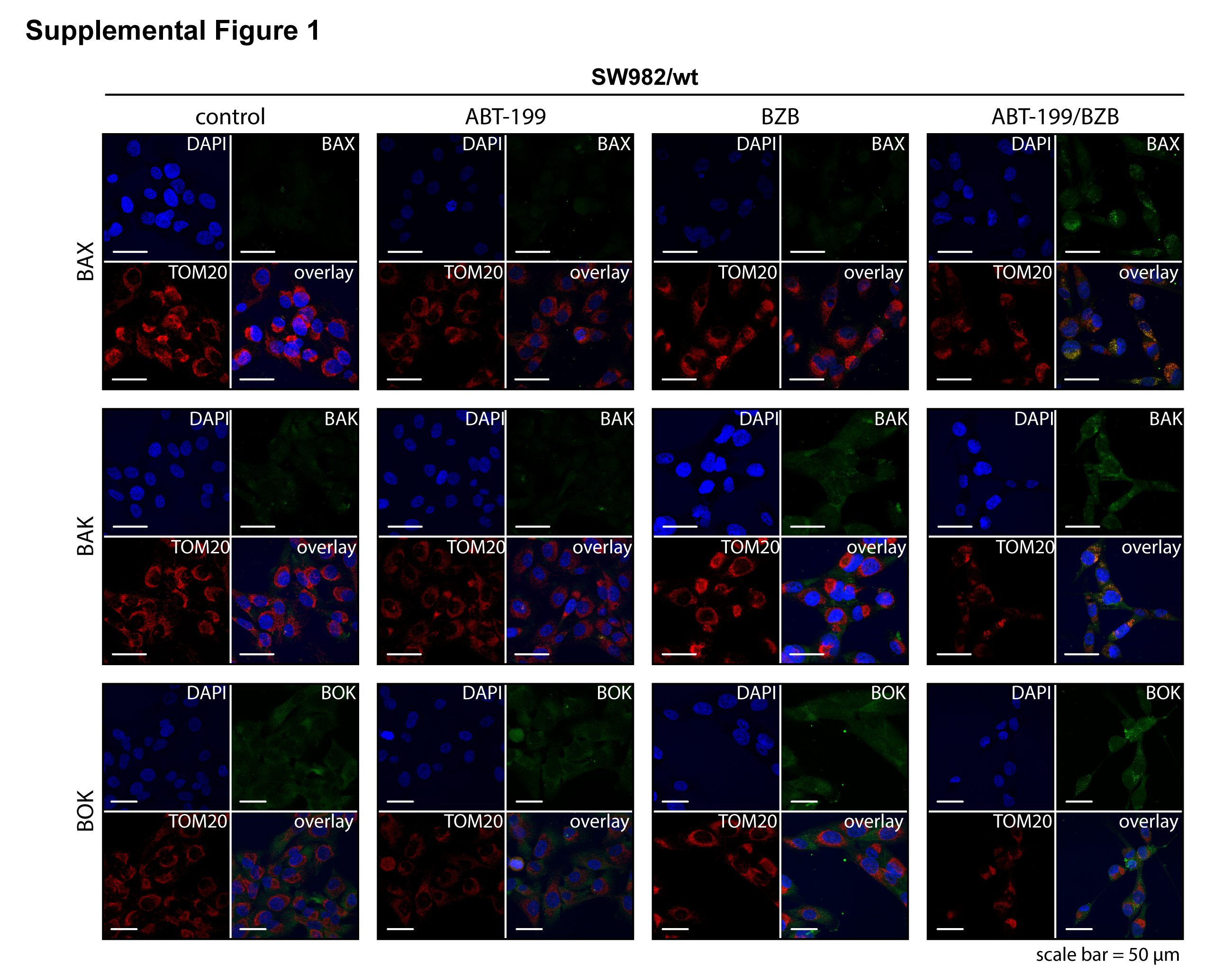

Supplement: Supplementary file 2 — Supplemental Figure 1 [file 41419_2020_2910_MOESM2_ESM.tif]

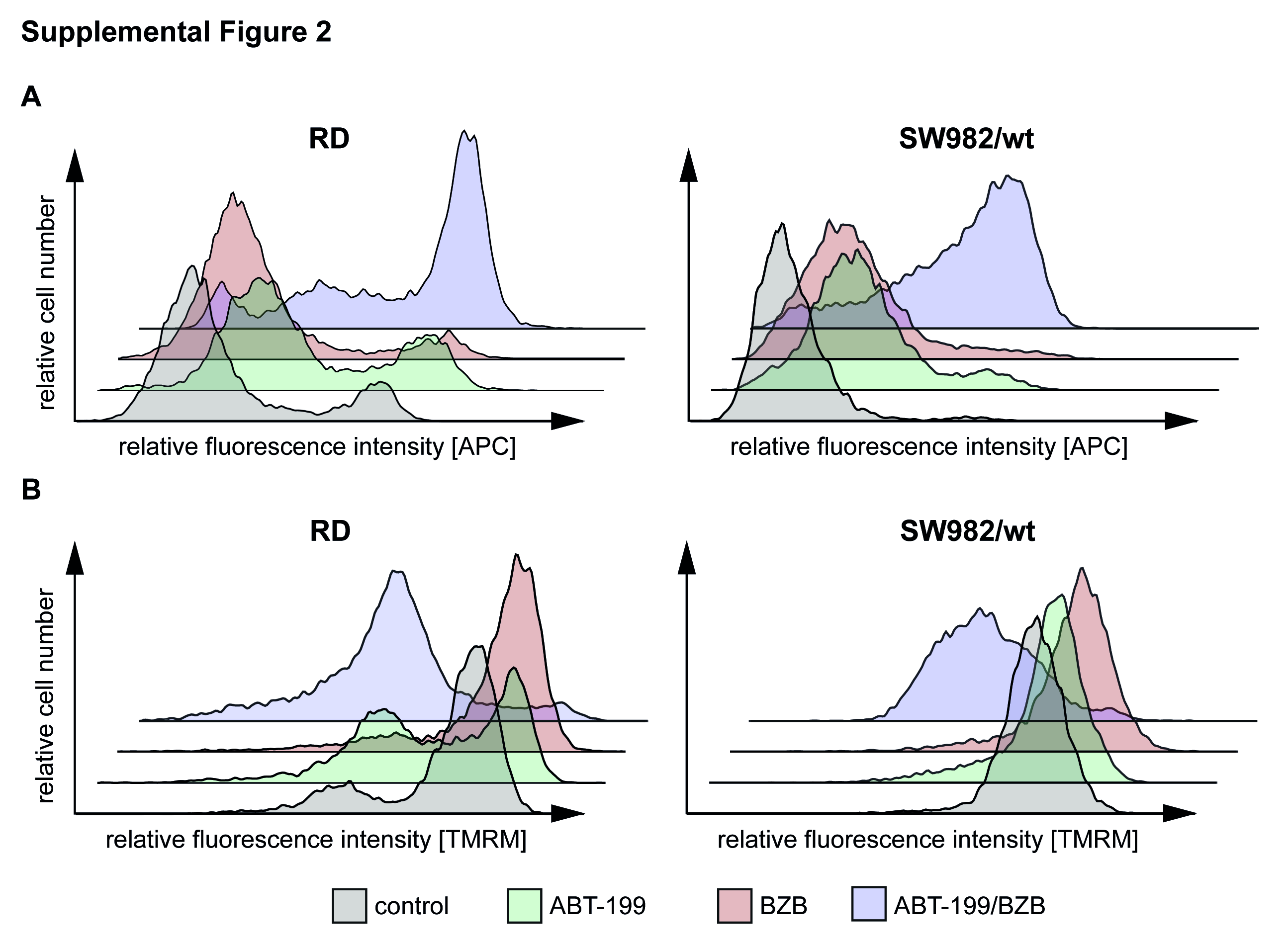

Supplement: Supplementary file 3 — Supplemental Figure 2 [file 41419_2020_2910_MOESM3_ESM.tif]

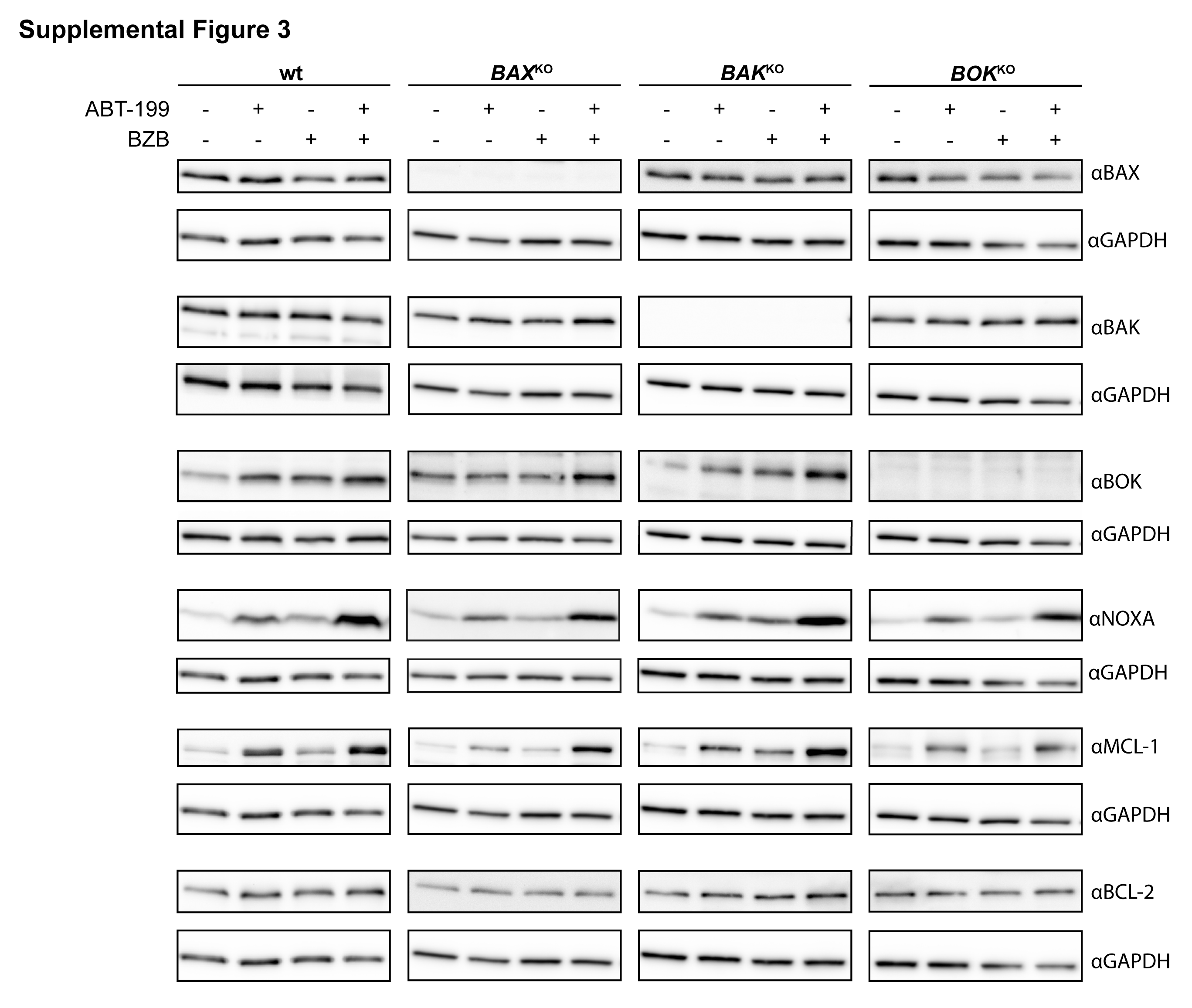

Supplement: Supplementary file 4 — Supplemental Figure 3 [file 41419_2020_2910_MOESM4_ESM.tif]

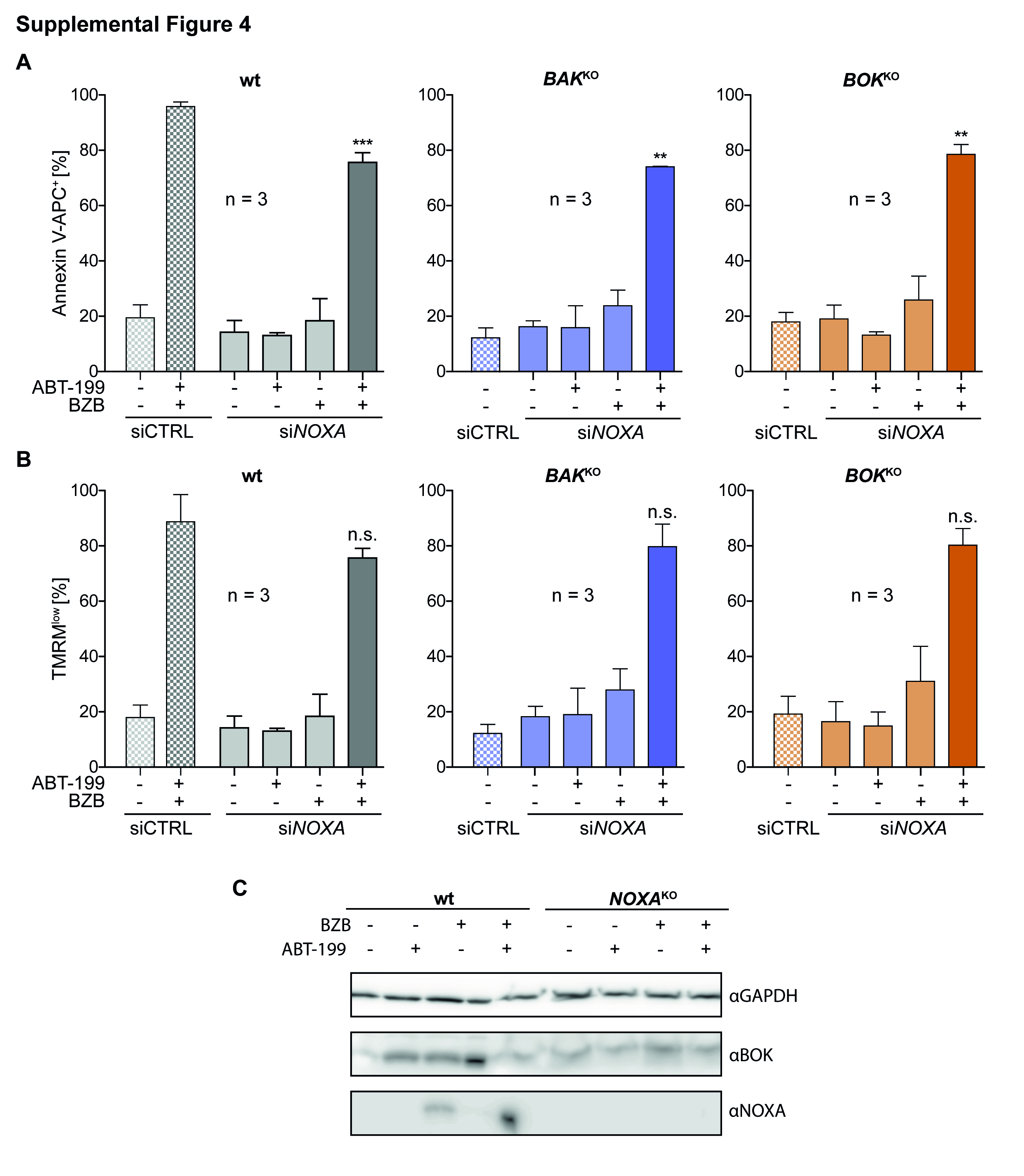

Supplement: Supplementary file 5 — Supplemental Figure 4 [file 41419_2020_2910_MOESM5_ESM.tif]

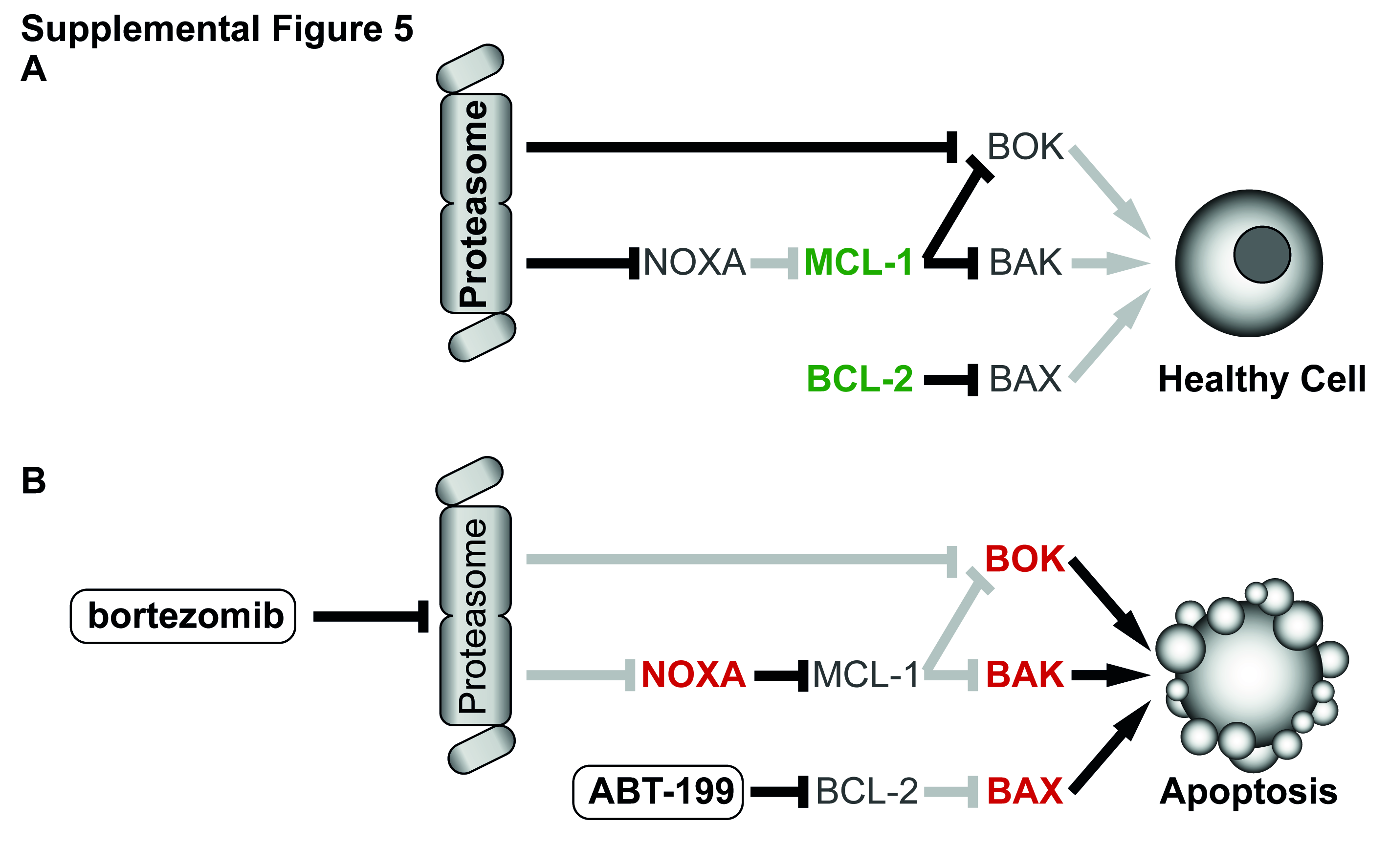

Supplement: Supplementary file 6 — Supplemental Figure 5 [file 41419_2020_2910_MOESM6_ESM.tif]
