# Supplementary material for: The BCL-2 selective inhibitor ABT-199 sensitizes soft tissue sarcomas to proteasome inhibition by a concerted mechanism requiring BAX and NOXA
Source: Cell Death Dis. 2020 Aug 24;11(8):701. doi: 10.1038/s41419-020-02910-2 (PMC7445285; doi:10.1038/s41419-020-02910-2)
Supplement: Supplementary file 7 — Supplemental Tables 1&2 [file 41419_2020_2910_MOESM7_ESM.docx]

**Supplemental tables**

**Supplemental Table 1: Soft-tissue sarcoma (STS) cell lines**

| **Cell line** | **STS subtype** | **Karyotype** | **Known mutations** | **ATCC® No.** | **Culture Medium** |
| --- | --- | --- | --- | --- | --- |
| SW982 | Synovial sarcoma (SS) | hyperdiploid | BRAF p.V600E Het. CDKN2A p.0? Hom. | HTB-93™ | DMEM |
| SK-LMS | Leiomyosarcoma (LMS) | aneuploid | TP53 G245S Het. | HTB-88™ | DMEM |
| SW872 | Liposarcoma (LS) | hypertriploid | BRAF p.V600E Het.  CDKN2A p.R80 Hom.  PTEN p.0? Hom.  TP53 p.I251N Hom. | HTB-92™ | DMEM |
| RH30/RD | Rhabdomyosarcoma (RMS) | unstable | NRAS p.Q61H Hom.  TP53 p.R248W Hom. | CCL-136™ | DMEM |
| SW1353 | Chondrosarcoma (CS) | hyperdiploid | nd | HTB-94™ | DMEM |
| Sa-OS | Osteosarcoma (OS) | hypotriploid | TP53 p.0? Hom.  Rb1 p.? Hom. | HTB-85™ | DMEM/F-12 |

**Supplemental Table 2: Primary sarcoma cell lines**

| **Cell line** | **STS subtype** | **Gender** | **Age** | **Histopathological Grade (1-3)** | **Culture Medium** |
| --- | --- | --- | --- | --- | --- |
| P-SS | Synovial sarcoma (SS) | F | 55 | 2 | DMEM |
| P-LMS | Leiomyosarcoma (LMS) | M | 75 | 3 | MEM |
| P-LS | Liposarcoma (LS) | M | 76 | 3 | DMEM |
| P-RMS | Rhabdomyosarcoma (RMS) | F | 25 | 3 | MEM |
| P-CS | Chondrosarcoma (CS) | M | 49 | 3 | MEM |
| P-OS | Osteosarcoma (OS) | M | 13 | 3 | DMEM |
